# Supplementary material for: Space Analysis of School Violence in the Educational Setting of Peru, 2019
Source: Int J Environ Res Public Health. 2022 Nov 30;19(23):16044. doi: 10.3390/ijerph192316044 (PMC9736996; doi:10.3390/ijerph192316044)
Supplement: Supplementary file 1 [file ijerph-19-16044-s001.zip › ijerph-2002538-supplementary.pdf]

International Journal of
Environmental Research and Public Health

MDPI

Supplementary Materials

Table S1. Provincial distribution by area of residence according to the spatial analysis of psychological, verbal, and sexual violence in the IEBR 2019.

Departments	Total number of provinces	Physics		Psychological		Sexual	
		Urban	Rural	Urban	Rural	Urban	Rural
**Amazonas**	5	NI	NI	NI	NI	1	1
Ancash	18	NI	NI	1	NI	NI	NI
Apurímac	7	**1**	NI	NI	NI	NI	NI
Arequipa	5	NI	NI	1	NI	NI	NI
Ayacucho	7	NI	**1**	NI	NI	2	1
Cajamarca	10	NI	NI	NI	NI	1	NI
Callao	1	NI	NI	NI	NI	NI	NI
Cusco	11	NI	NI	NI	NI	NI	NI
Huancavelica	6	NI	NI	NI	NI	NI	NI
Huánuco	8	NI	NI	NI	NI	NI	1
Ica	5	NI	NI	NI	NI	NI	NI
Junín	7	NI	NI	1	NI	NI	NI
La libertad	6	NI	NI	NI	NI	NI	NI
Lambayeque	3	NI	NI	NI	NI	NI	NI
Lima	8	NI	NI	NI	1	NI	NI
Loreto	5	NI	NI	NI	NI	NI	NI
Madre de Dios	3	NI	NI	NI	NI	NI	NI
Moquegua	2	1	NI	NI	NI	1	NI
Pasco	2	NI	NI	NI	NI	NI	NI
Piura	7	NI	NI	NI	NI	NI	NI
Puno	8	NI	NI	NI	NI	NI	NI
San Martin	8	NI	NI	NI	NI	2	1
Tacna	4	1	3	1	3	NI	NI
Tumbes	3	NI	NI	NI	NI	NI	NI
Ucayali	3	NI	NI	NI	NI	NI	NI

NI = unidentified.
